# Design and Analysis of a Differential Waveguide Structure to Improve Magnetostrictive Linear Position Sensors

**DOI:** 10.3390/s110505508

**Published:** 2011-05-20

**Authors:** Yongjie Zhang, Weiwen Liu, Haibo Zhang, Jinfeng Yang, Hui Zhao

**Affiliations:** School of Electronic, Information and Electrical Engineering, Shanghai Jiao Tong University, Shanghai 200240, China; E-Mails: zhangyongjie@sjtu.edu.cn (Y.Z.); weiwenliu@sjtu.edu.cn (W.L.); haibozhang@sjtu.edu.cn (H.Z.); yangjinfeng@sjtu.edu.cn (J.Y.)

**Keywords:** linear position sensors, magnetostrictive device, differential waveguides

## Abstract

Magnetostrictive linear position sensors (MLPS) are high-precision sensors used in the industrial field for measuring the propagation time of ultrasonic signals in a waveguide. To date, MLPS have attracted widespread attention for their accuracy, reliability, and cost-efficiency in performing non-contact, multiple measurements. However, the sensor, with its traditional structure, is susceptible to electromagnetic interference, which affects accuracy. In the present study, we propose a novel structure of MLPS that relies on two differential waveguides to improve the signal-to-noise ratio, common-mode rejection ratio, and accuracy of MLPS. The proposed sensor model can depict sensor performance and the relationship of sensor parameters. Experimental results with the new sensor indicate that the new structure can improve accuracy to ±0.1 mm higher than ±0.2 mm with a traditional structure. In addition, the proposed sensor shows a considerable improvement in temperature characteristics.

## 1. Introduction

Linear position sensors based on magnetostrictive effect are widely used in the industry today for position measurement and monitoring. In accordance with the Wiedemann effect and the Villari effect, the magnetostrictive linear position sensor (MLPS) uses a ferromagnetic material waveguide to perform accurate position measurements [1,2]. In comparison with other types of sensors, the MLPS is preferred for its accuracy, reliability, and cost-efficiency in performing non-contact, high precision, and long-range measurements [3–5]. A MLPS allows measurement without contact between the cursor and the sensing rod; thus, the device can have a long service life and a high level of ingress protection under harsh industrial conditions [6]. However, there are also disadvantages in the use of MLPS caused by their high-speed time measurement. In fact, even minor electromagnetic interference (EMI) or noise can lead to great measurement errors and increasing the signal-to-noise ratio is the key design issue for MLPS.

Previous attempts have been made to increase sensor accuracy such as exploiting the interference of undamped echoes and controlling the excitation period as described by Ferrari [7]. Hristoforou arranged two receiving coils at both ends of the sensor to obtain a better level of position sensitivity [8]. In the present paper, we illustrate a novel differential waveguide structure that can improve the signal-to-noise ratio and accuracy of the sensor. The proposed structure has been patented to prreserve the authors’ rights on the use of the device.

## 2. Principle of the MLPS

**Figure 1.**
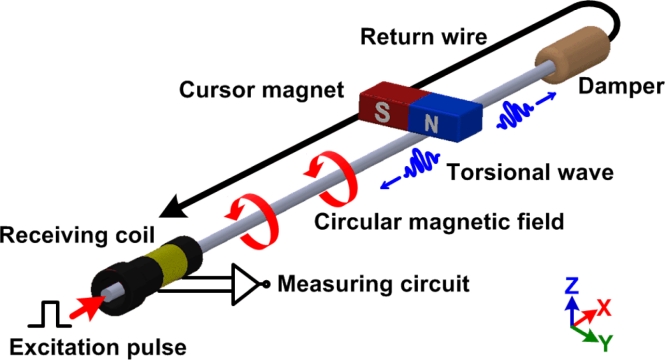
Principle of MLPS.

The principle of the MLPS is illustrated in Figure 1. The emitter periodically generates an excitation pulse through a ferromagnetic material waveguide, producing a circular magnetic field around it [9].

**Figure 2.**
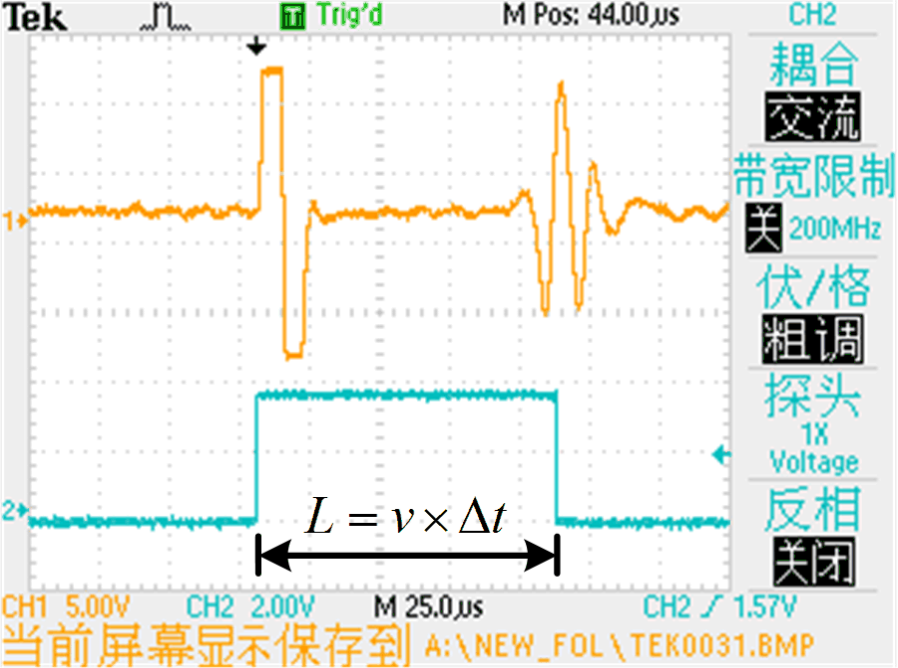
Oscilloscope waveform of induction signal.

The interaction between the magnetic fields due to the cursor magnet and excitation pulse causes a rotation of the magnetic domains in the waveguide [10]. According to the Wiedemenn effect, two torsional waves are induced in the waveguide away from the cursor magnet in both directions at a certain speed. The coil at one end of the waveguide acts as the receiver while the other end connects to the damper. The damper attenuates the torsional wave to avoid reflecting it back and corrupting at the other end. When the torsional wave passes under the receiving coil, the flux lines of the residual magnetic field change [11–15]. As described by the Faraday-Lenz Law, the change in permeability induces a voltage variation at the receiving coil output. The cursor position can then be computed from the propagation time as the torsional wave travels from the cursor magnet to the receiving coil. An example of the electrical waveform at the receiving coil is shown in Figure 2.

If we get the propagation time Δ*t*, with the knowledge of the propagation speed *v*, the position *L* is computed as:

(1) L=v×Δt

Based on electromagnetic effects, a MLPS is susceptible to EMI. Due to EMI, the instability of the induction signal will cause random errors in high-speed time measurements. The random error, which cannot be compensated for in the calibration process, is the main component that causes measurement uncertainty. To radically improve the SNR and sensory performance, we propose a different waveguide structure for the MLPS, which will be discussed in the next section.

## 3. Differential Waveguide Structure of Magnetostrictive Linear Position Sensor

**Figure 3.**
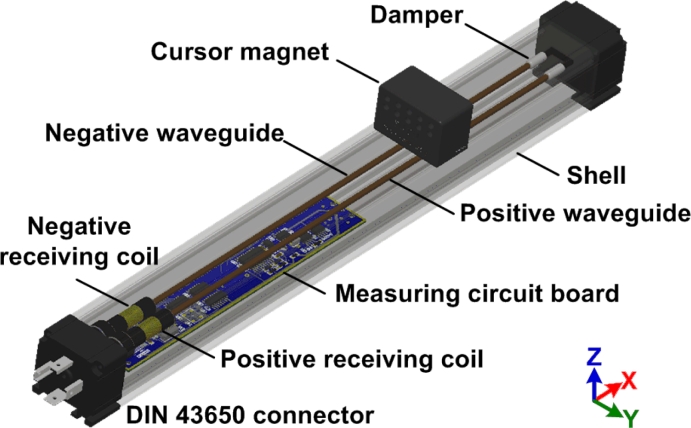
Proposed differential waveguide structure of MLPS.

The new structure of the magnetostrictive linear position sensor is explained in Figure 3. The most important features of the proposed MLPS are the presence of two waveguides and two receiving coils. The two waveguides (one positive and another negative) are made of the same material, have the same length, and they are connected by a wire at the distal end. Two receiving coils (one positive and another negative) are assembled at the proximal end with the same parameters.

**Figure 4.**
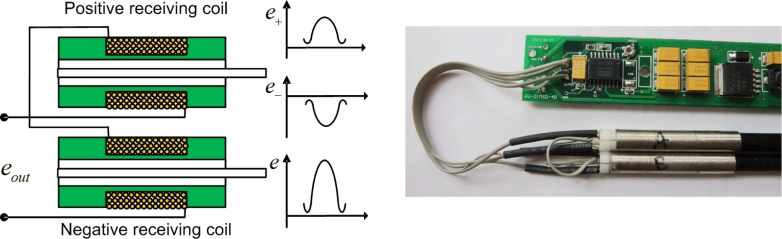
Structure with differential coils.

As shown in Figure 4, the positive and negative receiving coils are connected in a head-to-tail position at the measurement circuit in order to form a differential structure. The electronic signal inducted by the positive receiving coil is opposite to the negative receiving coil because the direction of exciting current through two receiving coils is opposite. Consequently, the signal intensity and signal-to-noise ratio of the new sensor is in theory doubled from those in the traditional single-mode waveguide sensor.

## 4. Sensor Model

**Figure 5.**
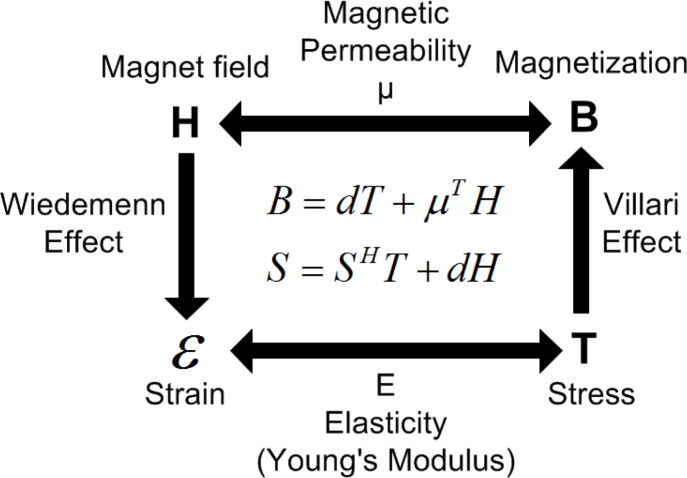
Magnetomechanical coupling in MLPS.

To analyze the performance of the proposed differential waveguide structure sensor and depict the relationship of the parameters of MLPS, we have built a sensor model including an analysis of several effects, as shown in Figure 5.

### 4.1. Wiedemenn Effect Model

When the exciting pulse arrives at the cursor magnet, the combination of a longitudinal magnetic field and a circular magnetic field created by excitation current will cause a torsional oscillation in the waveguide [16]:

(2) $$E=\frac{4\pi \lambda^{2}\mu }{k^{2}}$$

where *E* is Young’s modulus, *λ* is the magnetostrictive constant, *μ* is the reversible permeability, and *k* is the electromechanical coupling coefficient [17].

Since *F* = *Eɛ *= *E*Δ*s =λEA*, where *F* is the mechanical force, *ɛ* is the strain, and *A* is the cross-sectional area of the waveguide, Equation (2) can thus be rewritten as:

(3) $$F=\frac{4\pi \mu A\lambda^{3}}{k^{2}}$$

### 4.2. Villari Effect Model

Magnetostrictive materials convert magnetic energy to mechanical energy and *vice versa.* When propagating, the torsional wave causes the change in permeability of the waveguide [18–20]:

(4) $$\mu =\frac{M_{s}^{2}}{2K+3\lambda Eɛ}$$

where *μ* is the permeability, *M_s_* is the saturation magnetization, and *K* is the magnetic anisotropy constant.

Furthermore, *Eɛ* = σ = *F/ A* = *ma/ A*, where σ is the stress, *m* and *a* are the mass and acceleration associated with the mechanical force, respectively. Equation (4) can be rewritten as:

(5) $$\mu =\frac{M_{s}^{2}A}{2KA+3\lambda F}$$

Equation (5) shows the relationship between the mechanical force and the change in permeability of the sensing material.

### 4.3. Faraday-Lenz Law Model

The local flux line of the residual magnetic field is changed by the torsional wave. When the torsional wave passes under the receiving coil, the coil will have a voltage output [21,22]:

(6) $$\nabla \times H=J+\frac{dD}{dt}$$

(7) $$e_{0}=-N\frac{d\Phi }{dt}$$

where *e*_0_ is the induced voltage, *N* is the turns of the receiving coil, and Φ is the magnetic flux through a single loop. Besides, Φ = *BS* = *μSH* . Thus, Equation (7) can be rewritten as:

(8) $$e_{0}=-NS\frac{HvM_{s}^{2}k^{2}}{2L_{0}\;(KAk^{2}+6\pi \mu \lambda^{4})}$$

where *S* is the cross-sectional area of the receiving coil, *L*_0_ is the length of the receiving coil, and *v* is the velocity of the torsional wave.

The above three models are combined to predict the response of the MLPS. These predictions are compared to the experimental data. The percent difference of the actual measured data *versus* the predicted model is about 30%. We can consider the error as the energy loss when propagating on the waveguide. The sensor model could provide supplementary assistance when we design a MLPS.

### 4.4. Performance Analysis of the Differential Waveguide Structure of MLPS

We define the output of the positive receiving coil *e_+_* and the output of the negative receiving coil *e*___ in the following expression:

(9) $$e=e_{+}-e_{-}=-2NS\frac{HvM_{s}^{2}k^{2}}{2L_{0}\;(KAk^{2}+6\pi \mu \lambda^{4})}=2e_{0}$$

From the above expression, differential waveguide structures can double the signal intensity and increase the sensor sensitivity. Most of the interferences of MLPS, such as EMI, power ripples, and vibration noise, fall in the common-mode interference category. The differential waveguide structure of MLPS has an excellent common-mode rejection ratio. The accuracy of the sensor improves considerably with the improvement in anti-interference ability of the sensor.

## 5. Experimental Results

**Figure 6.**
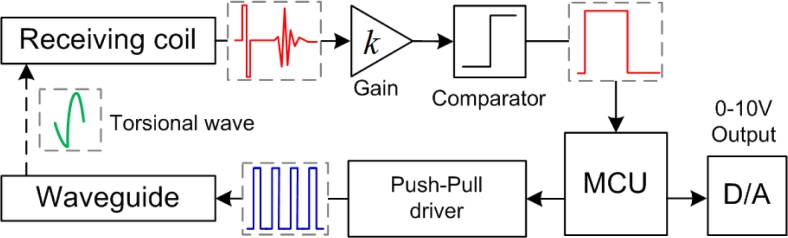
Block diagram of the circuit solution.

The block diagram of the circuit solution is shown in Figure 6. The MCU controls the Push-Pull driver to emit the excitation pulse periodically. After the receiving coil captures the torsional wave, then the induction signal is amplified by the high-speed instrumentation amplifier. A reference voltage V*_ref_* is compared with the amplified signal. As a consequence, the comparator produces a PWM signal proportional to the time interval between the excitation current and the torsional wave. MCU measures the time interval and provides a DC voltage proportional to the magnet position with a Digital-to-Analog converter and an operational amplifier. The direct position output, 0–10 Vdc, is widely used in the industrial field, thus the sensor is very convenient for installation and application.

**Figure 7.**
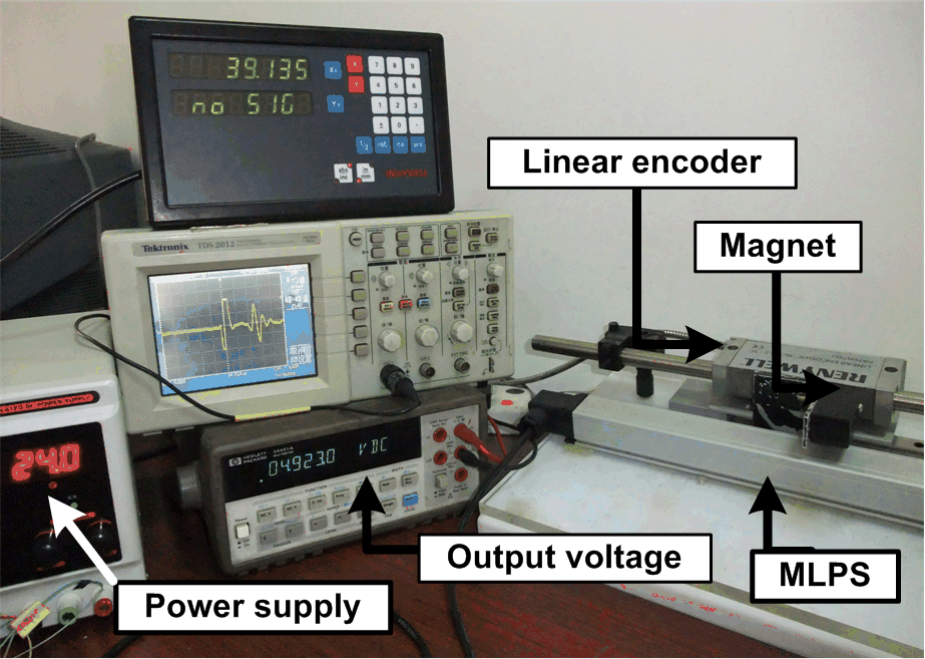
Experimental setup.

The experimental MLPS is shown in Figure 7. The device has been fixed to an immobile bench which also holds a rail with a moving tower. The cursor magnet is attached to the moving part and can move above the sensor. The placement of the cursor magnet can be measured by means of a linear encoder with a resolution of 5 μm. Through the experiment, the vertical distance between cursor magnet and waveguide remains invariant. The cursor magnet only moves in a measuring direction.

We made two magnetostrictive linear position sensors with 300 mm measurement range. One was a single-mode waveguide structure while the other one was a differential waveguide structure. Then, we compared the two sensors in the same experimental conditions listed in Table 1.

### 5.1. Comparison of Signal Intensity

**Figure 8.**
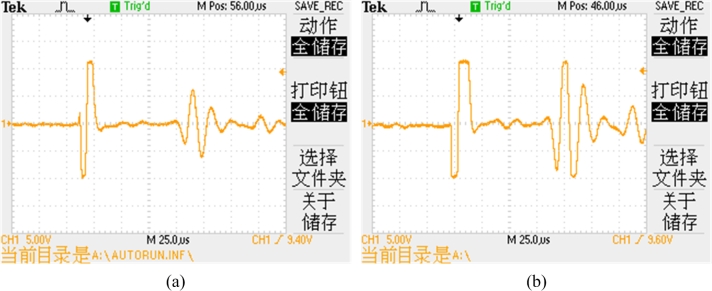
Waveforms of induction signal **(a)** with the single-mode waveguide structure; **(b)** with the differential waveguide structure.

Figure 8(a,b) shows the oscilloscope waveforms of the induction signal with the single-mode waveguide structure and differential waveguide structure, respectively. The signals are received by the receiving coils and amplified with the same parameters. The signal amplitude with differential waveguide structure is about twice in size of the single-mode waveguide structure. With this result, the differential waveguide structure can obtain an ideal induction signal with a lower amplification ratio. Therefore, the new structure improves the signal-to-noise ratio and decreases the influence by external noise.

### 5.2. Comparation of CMRR and Linear Precision

**Figure 9.**
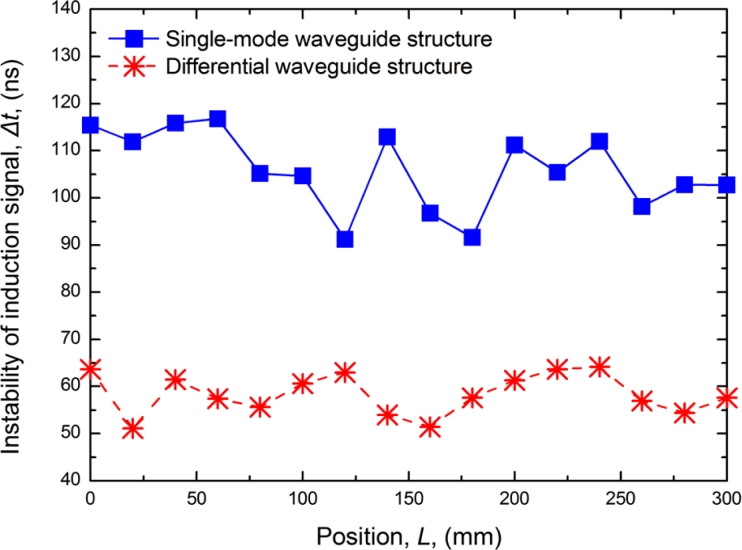
Comparison of induction signal instability.

After the calibration, the nonlinearity of the MLPS is determined by the instability of the induction signal. The anti-interference performance is very important. Induction signal instability is related to external EMI, power ripples, amplifier noise, and torsional reflected waves. With the differential waveguide structure, the signal instability calculated by the counter value fluctuation in the processing unit is reduced from 100 to 60 ns using traditional structure sensors, as shown in Figure 9.

**Figure 10.**
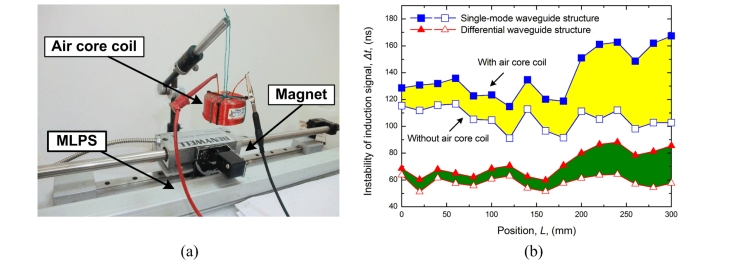
CMMR test with the air core coil **(a)** Experimental setup; **(b)** Comparison of induction signal instability with the perturbation.

In a further evaluation by CMMR, we set an air core coil above the sensor and position magnet. An excitation signal consisting of a settled sine train created by an arbitrary function generator (Tektronix AFG310) flows through the air core coil, as shown in Figure 10(a). This movement can guarantee that the electromagnetic perturbation induced by the air core coil is invariant [23,24]. When the air core coil is halfway through the measurement range, we move the position magnet to complete the full scale measurement and get the induction signal instability curve, as shown in Figure 10(b). When the air core coil approaches the receiving coil, a greater perturbation is induced. When the interferential coil is near or after the position magnet in measurement direction, less perturbation will be induced due to the magnetic field interaction and damping. The increment of instability with differential structure is smaller than the single-mode waveguide structure. Therefore, a higher CMRR could be obtained with the differential structure.

**Figure 11.**
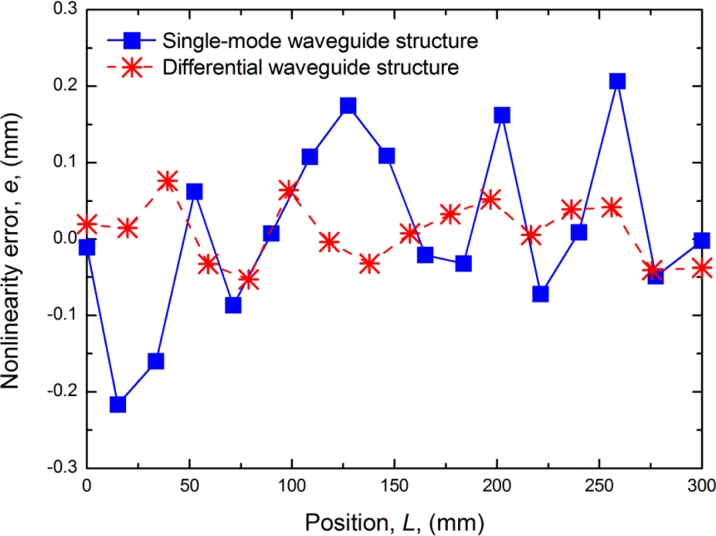
Comparison of linear error.

The experimental results of the linear error shown in Figure 11 indicate that the new structure can improve accuracy to ±1.1 mm, better that the ±0.2 mm achievable with a traditional structure. Apparently, a higher accuracy is obtained with the differential waveguide structure. Accuracy can still be further improved if the differential structure were manufactured with better symmetry.

### 5.3. Comparison of Temperature Drift

**Figure 12.**
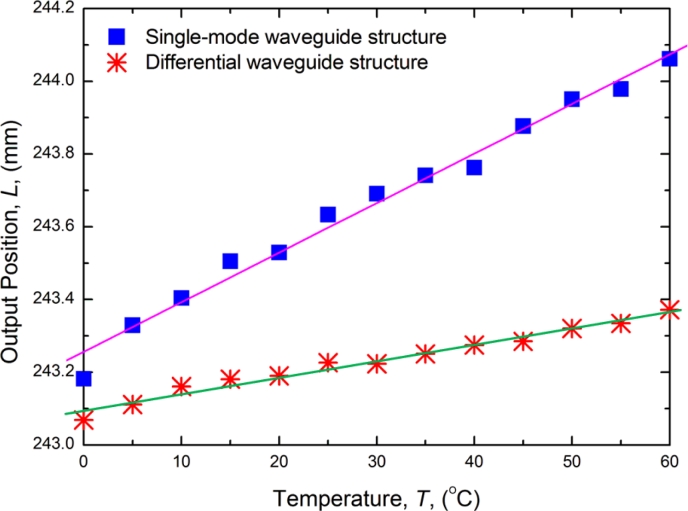
Comparison of temperature drift curve.

Temperature drift is one of the most important static characteristics of sensors. The temperature experiments were performed using a temperature control chamber. The temperature was raised from 20 to 60 °C at 5 °C intervals and each temperature point was maintained for 15 min. Figure 12 shows the comparison in terms of temperature drift between the single-mode waveguide structure and the differential waveguide structure. The results show that the temperature drift was smaller with the differential waveguide structure.

**Figure 13.**
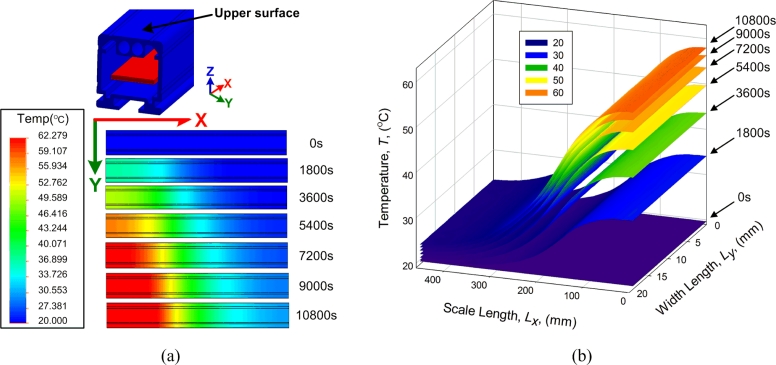
Finite element method simulation on heat conduction of the sensor in 3 h. **(a)** Temperature change on the upper surface of the shell; **(b)** Temperature data on the upper surface.

The working temperature of the sensor affects the amplitude of the induction signal generated by the receiving coil. For Ni-span-C magnetostrictive wire, the thermal expansion is 5 ppm/°C, the propagation velocity change is 3 ppm/°C, and the magnetostrictive constant is only 30 ppm. The effect is significant and must be taken into account where precision measurement is required [25,26]. The performance of the thermal conduction is investigated with a finite element method program. During the simulation process, the measurement circuit works at 60 °C and the environmental temperature was 20 °C. The temperature changes within 3 h as indicated in Figure 13(a).

In Figure 13(b), the rate of temperature change becomes slower with time. In the end, the sensor shows a temperature balance. In the measuring direction (X direction), there is a large temperature difference that causes a nonlinear sensor error. Although the error could be compensated for in the calibration process, the actual working temperature could not be compensated in advance. This is an important problem that requires resolution. Moreover, the temperature variation in Y direction is comparatively small; therefore, the thermal effects to the two waveguides may be considered the same due to the short distance. The differential structure compensates for the error caused by thermal expansion. In summary, the differential waveguide structure improves the temperature performance as well. In order to obtain more essential theoretical support from the experimental result, the temperature characteristics of the differential structure MLPS will be expounded in a future study.

## 4. Conclusions

In the present paper, we have proposed a new structure for a magnetostrictive linear position sensor that contains two waveguides. The new MLPS model could predict performance with certain precision. The new differential waveguide structure sensor provides higher signal-to-noise ratio, common-mode rejection ratio, and linear precision than the traditional single-mode waveguide structure. In addition, the present study provides a practical and reliable implementation technique for the commercialization of the MLPS.

## Figures and Tables

**Table 1.** Experimental conditions for MLPS.

**Item**	**Parameter**	**Symbol [Unit]**	**Material, Value**
Magnetostrictive wire	Material		Ni-Span-C
	Length	L_s_ [mm]	300
	Diameter of wire	d [mm]	0.5
	Torsional modulus	E [kN mm^−2^]	70
	Resistance	R [Ω]	3.6

Pulse current	Amplitude	I_p_ [A]	3
	Period	T_w_ [ms]	1
	Width of pulse	T_p_ [μs]	5

Cursor magnet	Material		BaFe_12_O_19_
	Size	[mm]	20×30×8

Amp.	Instrument Amplifers	[Av dB]	49.5 + 20

Receiving coil	Number of turns	N [turn]	800
	Length	L_c_ [mm]	5.2
	Wire diameter	d_w_ [mm]	0.05

Torsional wave	Speed	v [m s^−1^]	2650
